# Choroidal vascularity index is independent of ocular and image-based factors in healthy eyes: a systematic review and meta-analysis

**DOI:** 10.1038/s41598-025-10384-5

**Published:** 2025-07-30

**Authors:** Meenakshi Kumar, Sieu K. Khuu, Matt Trinh, Michele C. Madigan, Rupesh Agrawal, William Rojas-Carabali, Lisa Nivison-Smith

**Affiliations:** 1 School of Optometry and Vision Science, University of New South Wales (UNSW Australia), Sydney, NSW 2052 Australia; 2 Centre for Eye Health, University of New South Wales, Sydney, 2052 Australia; 3 National Healthcare Group Eye Institute, Tan Tock Seng Hospital, Singapore, Singapore; 4 Lee Kong Chian School of Medicine, Nanyang Technological University, Singapore, 308433 Singapore; 5 Singapore Eye Research Institute and Singapore National Eye Centre, Singapore, Singapore; 6 Duke NUS Medical School, Singapore, Singapore

**Keywords:** Normative value, CVI, Choroid, Vessel lumen, Health care, Medical research

## Abstract

Choroidal vascularity index (CVI) has been extensively used to assess choroidal health in posterior eye disease; however, interpretation is hindered by conflicting evidence on effect of normal physiological factors on CVI. Thus, this review aimed to derive a normative value for CVI and understand its relationship with various normal ocular/image-based factors. Studies were screened for eligibility, defined as eyes with normal ocular parameters (refractive error (RxE) < ± 6 DS, intraocular pressure (IOP < 21 mmHg) and no posterior segment disease and 98 studies were included. Pooled weighted average of subfoveal CVI extracted from healthy eyes (n = 5332 eyes) was 66.50% [CI 65.67–67.32]. Secondary stratifications (time of scan, device wavelength, imaging mode, region of interest, systemic factors) and meta regression (age, RxE and IOP) were insignificant on CVI (*p* = 0.579–0.872), however Best corrected visual acuity showed positive correlation (*p* = 0.037). Unaltered, narrow confidence interval in sensitivity analysis, complemented by non-significant publication bias indicated robustness of the synthesised data. These results provide the highest-level evidence in hierarchy of the first normative subfoveal CVI value synthesised from literature and that it is not influenced by most person, eye and imaging factors in healthy eyes.

**Supplementary Information:**

The online version contains supplementary material available at 10.1038/s41598-025-10384-5.

## Introduction

The choroid is the most vascularised structure of the eye and plays a crucial role in blood supply and metabolic support of the outer retina^1^, thermoregulation, ocular accommodation anteriorly, and modulation of scleral growth^2^. Not surprisingly, structural and function alterations in the choroid are considered to have a primary role in ocular diseases such as age-related macular degeneration (AMD)^3^, diabetic chorioretinopathy^4^ polypoidal choroidal vasculopathy (PCV)^5^, myopia^6^ and uveitis^7^. Assessing choroidal vascular health is thus is a key factor in understanding early ocular disease and ensuring appropriate disease monitoring.

The choroidal vascularity index (CVI) is a non-invasive measure of choroidal vascularity that is based on the ratio of the vascular area (LA) to the total choroidal area (TCA) from binarized image. CVI has been applied to numerous vascular diseases^8^ and found critical clinical insights including early detection of AMD^9^, glaucoma^10^, diabetes mellitus^11^ and central serous chorioretinopathy^12^. Population studies who explored this tool in normal healthy participants however are, either assessing only ethnically homogenous samples^13^, or lacking critical details in their definition of healthy population such as refractive error/axial length criteria or including participants with non-macular diseases^14^. The reported associations of normal physiological factors such as age are also conflicting wherein some studies show no effect^15–17^ while others showing varying age-related associations^18–20^. Likewise associations between axial length^6,21^, and diurnal variation to CVI are also conflicting^22–24^. Together, poor understanding of the normative range for CVI limits its clinical application as it is difficult to confirm if alterations in CVI in ocular disease are due disease pathology or just physiological variations.

Thus, this systematic review and meta-analysis aimed to compile and analyse the accumulated literature on CVI to determine

1. the pooled average normative choroidal vascularity index value in healthy individuals without posterior segment disease and,
2. the person, image and eye-specific factors that significantly influence this measure

By synthesising the highest level of evidence in research hierarchy, i.e. systematic review and meta-analysis, this study will provide essential baseline values that enhance the clinical applicability of CVI, improve its reliability as a biomarker for choroidal vascular health, and pave the way for future research in ocular disease assessment.

## Results

### Selection of studies

**Fig. 1:**
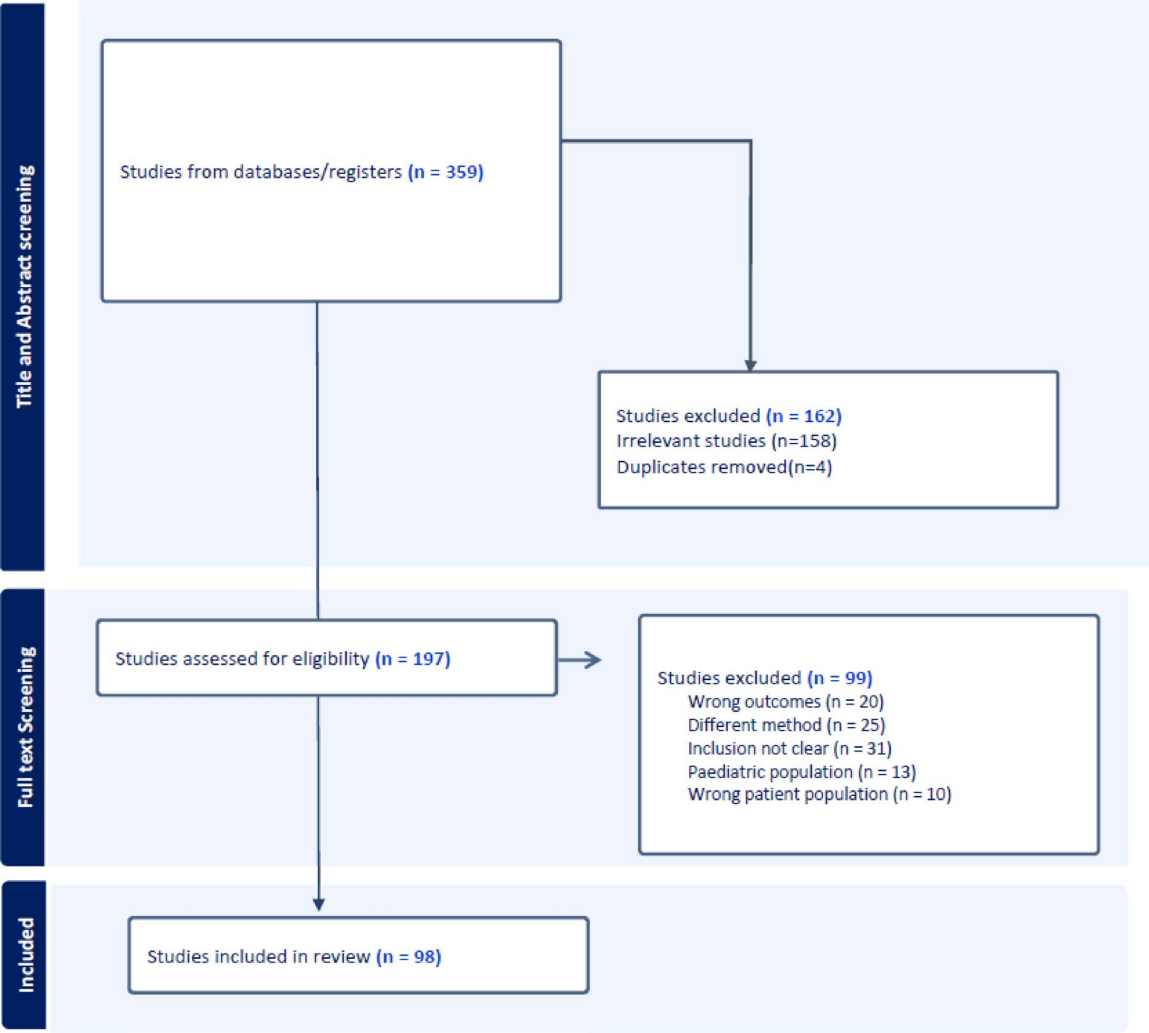
Prisma flowchart of screening process.

The electronic database search initially yielded 359 studies which was reduced to 197 studies after screening for title and abstract. Ninety-nine studies were further excluded after full text screening for reasons including wrong population, different method of CVI calculation, unclear inclusion criteria, paediatric population and wrong outcome (full list of reasons for exclusion in Supplementary Table 1). A total of 98 unique studies were eligible for the final review and meta-analysis (Fig. 1).

### Study characteristics and quality assessment

A detailed summary of all included studies is presented in Supplementary Table 2. All included studies were observational and cross-sectional in nature: 83 studies were case–control studies, and 15 studies were cross-sectional based either on assessment of healthy participant characteristics (effect of wearing masks, valvalsa manoeuvre on CVI, n = 6) or assessing CVI methodology in healthy subjects (differences in OCT machines, using AI for measurement of CVI etc., n = 8). The majority of studies were conducted in Turkey (42/98)^16,19,21,25–63^ followed by the UK (14/98)^20,64–76^, India (10/98)^77–86^, China (9/98)^87–95^, United stated (8/98)^96–103^, Singapore (5/98)^11,13,104–106^, Korea (5/98)^4,10,107–109^, Iran (2/98)^110,111^, Tokyo (1/98)^112^ and Australia (1/98)^18^. Sample size ranged from 6 to 345 eyes and average age of participants ranged from 22 to 77 years. Statements about funding and conflicts of interests are listed in Supplementary Table 3.

All the publication factors were reviewed for quality assessment. Most studies (91/98) had at least one section of high risk of bias (Supplementary Table 4). For the section, “Representative of the cohort”, 93% of the studies displayed high risk by having non-consecutive recruitment for the extracted group while 7% of studies recruited consecutive patients displaying low risk. For “Selection of the controls”, 4% of studies recruited the extracted group from the community and 72% recruited the group from a university or hospital population displaying low to moderate risk while 22% of studies did not provide adequate descriptions to determine a classification. In the section, “Definition of controls”, 82% of the control population had no history of disease indicating low risk, while 22% had an inadequate description of presence or absence of disease and were simply termed as *healthy population* displaying unclear risk. For the section “Ascertainment of exposure”, 72% of the studies had a prospective study design, and 25% had a retrospective study design displaying low risk. One study had an inadequate description of the study design indicating unclear risk.

### Quantitative assessment

#### Primary outcome (CVI)

**Fig. 2:**
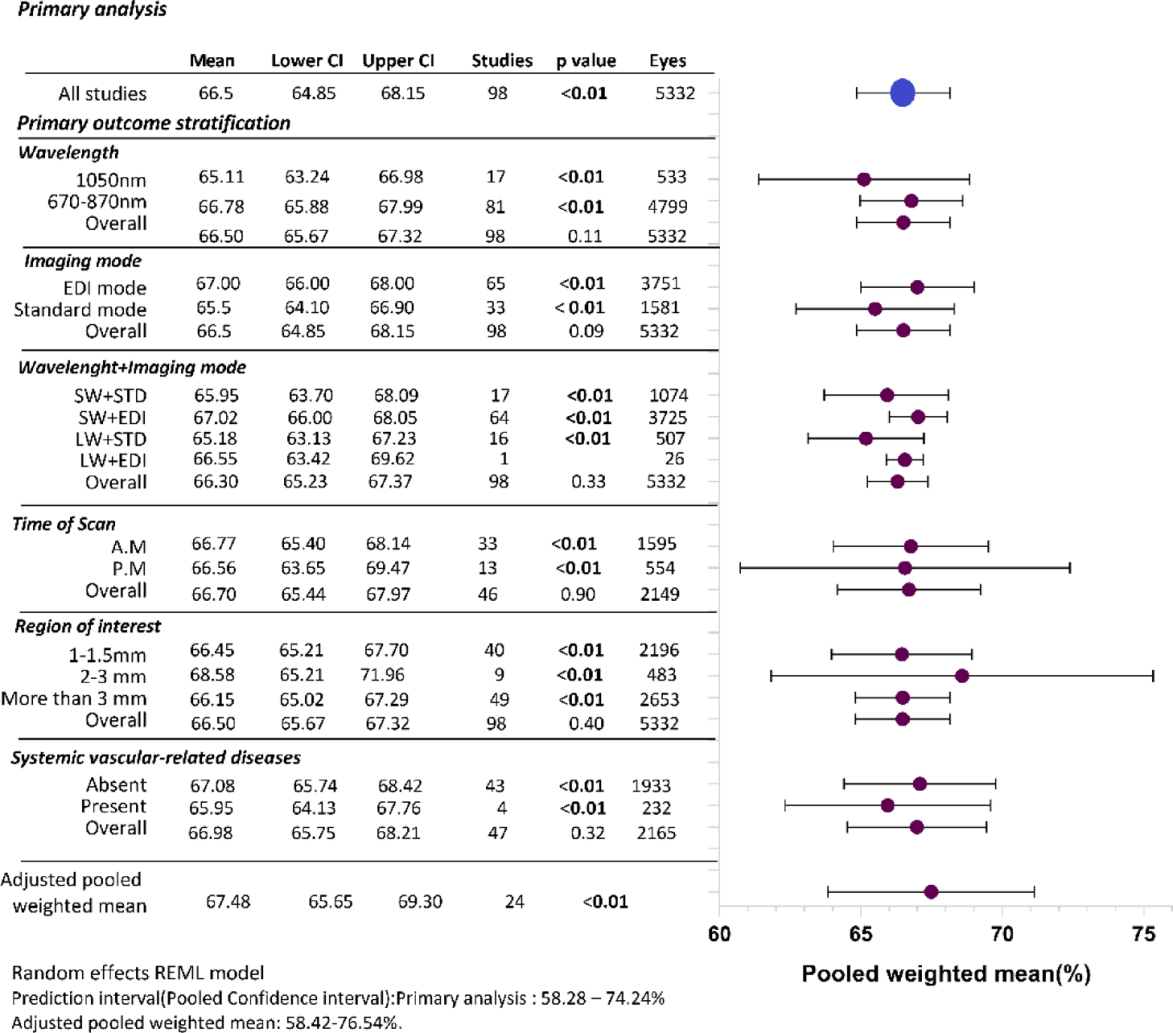
Results of primary outcome and stratification. The error bars indicate confidence intervals. *SW* short wavelength, *LW* long wavelength, *STD* standard imaging, *EDI* enhanced depth imaging.

All 98 studies were included in the primary meta-analysis. The pooled weighted average of subfoveal CVI of normal, healthy eyes (n = 5332 eyes) was 66.50% [CI 64.85–68.15] with a prediction interval of 58.28–74.24% representing the moderate heterogeneity of the data (Fig. 2, Supplementary Fig. 1).

#### Primary outcome stratifications

Data from 98 studies were collated to analyse the relationship of categorical image-level factors such as OCT device used, OCT imaging mode, time of day scan was taken and region of interest and individual level factors such as presence of systemic disease to CVI (Fig. 2).

#### Person-level factors

##### Presence of systemic disease

CVI was not significantly different between the 43 studies (n = 1933 eyes) that specified the absence of systemic disease in their population (67.08% [65.74–68.42%]) versus the 4 studies (n = 232 eyes) that reported a presence of systemic disease (diabetes/hypertension; 65.95% [64.13–67.76%], *p* = 0.62). Fifty-one studies (n = 3167 eyes) did not specify the presence or absence of systemic disease in a clear manner and were excluded from this stratification.

#### Image level factors

##### OCT device and scanning wavelength

All 98 studies reported the specific OCT devices used. Devices were then classified as longer wavelength OCT (1050 nm; i.e. Swept Source OCT DRI-OCT Triton OCT Topcon and VG200S; SVision Imaging, Henan, China, n = 17 studies, 533 eyes) or standard wavelength OCT devices (670–870 nm, Spectralis, Heidelberg Engineering GmbH, Heidelberg, Germany, Cirrus HD-OCT 5000, standard, Zeiss Cirrus, Topcon OCT-1000 Mark II, Software Version 4.21; Topcon Corp SD OCT Enhanced Depth Imaging mode, RTVue-XR Avanti, Optovue Inc., Fremont, CA, USA SD OCT, n = 81 studies, 4799 eyes). These devices have been shown to have good repeatability for choroidal measurements^93,113–118^. Weighted average of CVI from longer wavelength OCTs was 65.11% [63.24–66.98%] while weighted average of CVI of standard wavelength OCTs was slightly higher at 66.78% [65.88–67.69%]. The group effect size (θ) was individually significant (*p* = 0.000) however the overall group difference was statistically not significant (*p* = 0.11).

##### Time of scan (diurnal variation)

Forty-nine studies reported the time at which the OCT scan was acquired. Studies performed during *ante meridiem (AM)* (33 studies, 1595 eyes) has an average CVI of 66.77% [65.40–68.14%] which was not significantly different to those performed *post meridiem (PM)* (13 studies, 554 eyes) with CVI of 66.56% ([63.53–69.47%], *p* = 0.90). Three studies (211 eyes) reported time ranging from morning to evening and were not considered for this analysis.

##### Imaging mode

Sixty-five studies (n = 3751) used enhanced depth imaging (EDI) mode for capturing OCT B scans while 33 studies (1581 eyes) used the standard mode of imaging. CVI was higher for studies performed in EDI mode (67.00% [66.00–68.00%]) versus standard mode (65.60% [64.10–66.90%]) with the group θ individually significant (*p* = 0.00) but overall group difference not being statistically significant (*p* = 0.09).

##### Combination of OCT and imaging mode

Sixteen studies (507 eyes) used long wavelength OCT with standard imaging mode and reported CVI to be 65.18 ([63.13–67.23%], *p* = 0.00), while only one study reported using EDI mode (26 eyes) and the CVI to be 66.55 ([65.90–67.20%]). Similarly, studies using standard wavelength OCT with standard imaging mode (n = 17, 1074 eyes) reported CVI to be 65.95 ([63.70–68.09%], *p* = 0.00), while those who used EDI mode (n = 64, 3725 eyes) had a CVI value of 67.02 ([66.0–68.05%], *p* = 0.00). However, the overall group effect was not significant (*p* = 0.33).

##### Region of interest

All the studies defined the exact area over which CVI was quantified. Forty studies (n = 2196 eyes) calculated CVI within 1–2 mm of the fovea and reported a value of 66.45% [65.21–67.70%] while nine studies (n = 483 eyes) calculated CVI within 2–3 mm of the fovea, reporting a value of 68.59% [65.21–71.96%] and forty-nine studies (n = 2653 eyes) assessed an area of 3 mm up to the full scan area (~ 6–12 mm) and reported a CVI of 66.12% [65.65–67.32%]. The group difference in CVI was not significant between area sub-groups (*p* = 0.40).

##### Overall stratification

When assessed individually, the above factors didn’t show significant association with CVI, however when all the factors were analysed together, the adjusted CVI from twenty-four studies is 67.48% [65.80–69.30%] with the prediction interval ranging from 58.42 to 76.54%. The group difference was significant (*p* = 0.00, Fig. 2).

#### Meta regression

Data from 17 studies were collated for meta-analysis to analyse the relationship of continuous person level factors such as age, and eye-level factors such as axial length, BCVA, and IOP to CVI. Individually, these factors showed no association, however when all continuous person- and eye-level factors were tested together, BCVA showed a positive correlation with CVI within the specified normal limit of vision (Table 1).

**Table 1:** Ocular factors tested for association with CVI.

Tested variables	Coefficient	Std error	z	*P* >|z|	[95% Conf. interval]	
Age	− 0.030	0.055	− 0.550	0.579	− 0.137	0.077
BCVA(LogMAR)	6.513	3.116	2.090	**0.037**	0.405	12.620
IOP	0.137	0.848	0.160	0.872	− 1.526	1.800
Axial length	0.382	1.294	− 0.300	0.768	− 2.919	2.155

Significant values are in [bold].

Correlation with choroidal thickness:

CVI was found to form a weak linear relationship with choroidal thickness (n = 78, Mean CT: 285.5 ± 61.52 microns, Y = 2.778x + 101.9, R^2^ = 0.041) (Supplementary Fig. 2).

#### Sensitivity analysis

A sensitivity analysis was conducted to validate the primary outcome measure by adjusting specific circumstances such as exclusion of suspected shared populations and exclusion of studies which included both eyes in their sample size. The results revealed robustness of the data whereby neither the primary pooled weighted mean nor the prediction interval was not significantly altered (*p* = 0.69–0.91).

#### Publication bias

**Fig. 3:**
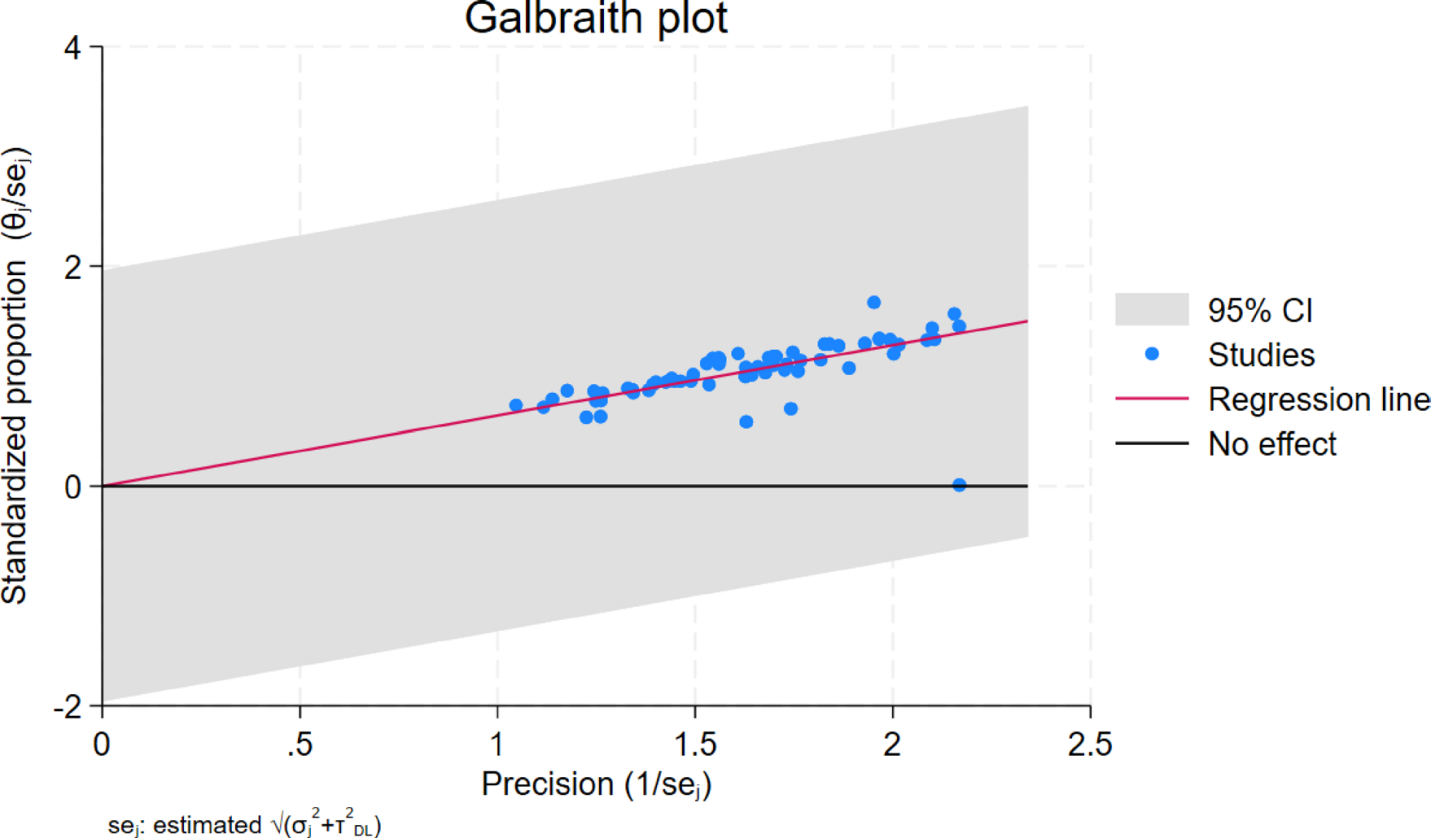
Galbraith plot showing studies within 95% confidence intervals.

Galbraith plot of the primary outcome demonstrated that 96% of the data fell within the 95% confidence interval estimation indicating low publication bias (Fig. 3).

#### GRADE assessment and summary of findings

The GRADE assessment concluded that the evidence from the collective studies in this review have moderate confidence that the described pooled weighted mean from the primary outcome is as stated (Supplementary Table 5).

## Discussion

This systematic review and meta-analysis found the weighted average CVI of a normal healthy population to be 66.50 ± 1.65% and independent of most person- and eye-level factors such as age, axial length, refractive error, and intraocular pressure when within a healthy range. When adjusted for image level factors, the pooled CVI increased to 67.48 ± 3.5% but device related variations such as area of measurement, excitation wavelength, imaging mode or time of day of scan had no significant influence. This review suggests that CVI is indeed a robust choroidal marker and could be compared against populations of healthy eyes even with variations in demographic or image acquisition characteristics.

This study found normal CVI was 66.5% suggesting almost two-thirds of the subfoveal choroid of healthy individuals is vascular lumen versus stromal tissue. This is similar to the estimates from *post mortem* eyes which report the vascular area of the choriocapillaris in the submacular region of normal eyes to be 51–80%^119,120^ (variation due to differences in study population, tissue processing, staining methodology and quantification methods). These findings are also consistent with reports of choroidal vessel density of healthy eyes using alternative analysis methods for OCT scans^121^ and choriocapillaris vascular density from more recent OCTA studies of normal healthy eyes (76–84%)^122–125^. Given that human choroid is reported to have the highest vascularity per unit weight^126^, this corresponds to the high luminal area vs stromal area reflected in CVI measurement ranges from 64 to 68.15% in a healthy population.

We did not observe a difference between CVI calculated from images acquired using “standard” wavelength (680–890 nm) and “longer” wavelength OCT (1050 nm). This contrasts previous studies that hypothesised that longer wavelength OCTs might provide more accurate CVI calculations because of increased choroidal visualisation due to deeper penetration and improved separation of lumen and stromal tissue^127^. We also did not find any effect of scan mode on CVI neither did combining OCT and imaging mode despite EDI mode being reported to increase choroidal visibility due to placement of the zero delay line^8^. The lack of impact from wavelength and scan mode could have been influenced (1) by device with most included studies (n = 63/98) using Spectralis OCT which already improves image resolution compared to standard wavelength OCT through differences in optics rather than wavelength^128,129^. Alternatively, in the process of binarization, the entire image is converted to absolute values of black and white, unifying subtle changes (2) considering imaging parameters that influence choroidal visibility such as the position of the zero delay line and the use of EDI are end-user dependent^130^, this may be an issue as 28/98 studies were retrospective and the intent of OCT capture could not be determined with certainty and lastly (3) the longer wavelength does indeed enhance the choroidal visibility, and that it does so equally for white and dark zones within the choroid, hence maintaining the proportionality within “the normal spectrum”. In a disease state, perhaps longer wavelenght might be better detect subtle changes in deeper choroid, additional research may be necessary to validate these findings. Studies utilising other imaging devices/modalities with lower resolution or studies with disease spectrum should take caution as the findings may not apply to them.

We also found no association between the time of day the scan was acquired and CVI despite choroidal circulation metrics being shown to undergo significant changes with the time of measurement^131^. Alanazi et al.^22^ and Kinoshita et al.^23^ previously reported changes in CVI within the same day but disagreed on the contributor to this variation (stromal vs non stromal). On the other hand, Singh et al.^24^ reported that the CVI of the temporal quadrant is impacted by diurnal variation while, macular CVI and other subfields are not. In this review, only 50% of included studies reported the time of day of scan acquisition and variability in reporting only allowed us to stratify studies into *AM* and *PM*. Thus, while no significant diurnal variation effect was found for CVI of healthy eyes, this may be due to the coarseness of categorisation and the incomplete data set. Future work is needed to confirm this hypothesis.

When all continuous eye-level factors were meta regressed together (IOP, refractive error, axial length, BCVA), only BCVA was significantly associated with CVI. BCVA has been shown to have linear correlation with CVI^88,132,133^. This is not surprising, given that cone-mediated vision has greater metabolic demands and requires at least a 15% higher choroidal blood flow compared to rod-mediated vision^134,135^, suggesting the use of CVI as an adjunctive test to monitor vision changes or disease progression. Although CVI is structural measure, there may be differences in hemodynamics which cannot be adequately characterised by CVI, flow changes can result in vasculature remodelling^136^ which can be reflected in CVI. Intraocular pressure, refractive error and axial length have previously been associated with CVI but this only appears to be relevant when these parameters are within the pathological range. For example, several studies have reported reduced CVI in myopia i.e. correlation between refractive error/axial length and CVI^25,84,137,138^. However, studies of refractive error within normative ranges (such as less than ± 6 diopters) have found no associations^13,15,21,37^. Wang et al.^132^ proposed a threshold where associations between refractive error and CVI are only observed above an axial length of 27.26 mm highlighting the complexity in the relationship. Thus, while CVI appears to be resistant to several ocular parameters, this effect appears to be only applicable within normative ranges and must be treated with caution with individual considerations.

Person-level factors such as including age, and systemic vascular diseases were not associated with CVI in healthy eyes when controlled for other factors. The literature indicates presence of diabetes mellitus^4,139^, hypertension^27^ and or cardiovascular disease^140^ impacts CVI. Although, a higher CVI was found in the absence of these conditions in this review, it did not reach statistical significance warranting further research. There has been a large amount of conflicting evidence regarding associations between age and CVI with several studies reporting a loss of CVI with age^18–20,84,85,137,141^ and others reporting no association^13,15–17,77,105^. Other OCT based choroidal parameters such as choroidal thickness normally decreases with age^142–145^ and evidence from *post mortem* studies strongly suggest the choroid undergoes thinning, choriocapillaris density decrease, vessel stenosis, loss of elasticity and composition changes in Bruch’s membrane with aging^146–148^. The choroidal area parameters that constitute CVI (TCA, LA and SA) also show age-related loss^15,18^. The null effect of age on CVI in this review suggests that a decline in luminal and choroidal area is proportional, at least in healthy eye^17,41^. This is supported by work of Ramrattan et al.^146^ who showed a linear decrease in capillary diameter and capillary density alongside total choroidal thickness over 10 decades of life. While we definitively cannot dimmish the findings of age association with CVI, this review provides highest evidence in hierarchy that under healthy considerations of BCVA, refractive error, IOP, axial length and limited systemic vascular related disease, CVI does not show association with age. As with imaging factors, these results should be interpreted with caution in disease populations with age-related diseases such as age-related macular degeneration where longitudinal changes in CVI have been reported^149^.

The main limitation in this review was the large number of studies were excluded because the candidate population was a control group within a case–control design that was poorly defined. These potential populations could not be adequately screened against our inclusion/exclusion criteria. Although, the risk of bias indicates a high score, we don’t believe that this limits the results. This is because of the nature of recruitment for control group, where in description of details is limited unlike the disease group. Studies which utilised alternative CVI methodology such as that described by Sonoda et al.^150^, were also excluded from this review to have consistent methodology. The field has evolved since the describe methodology and the method describe by Agrawal et al.^151^ is being predominantly used. Ocular magnification is known to play a significant role in structural components such as thickness and individual elements like total choroidal area, luminal area, stromal area. However CVI is a ratio of these structural measures and designed to mitigate inter-individual differences such as the effect of ocular magnification^15^. A study with similar transverse measurement indicated the corrected difference to be clinically insignificant^152^. Hence we believe the results to be unhampered in its application. The included studies also had poor reporting of factors such as ethnicity which could not be subsequently explored in meta-analyses. Despite this, a sample of 5332 eyes from 98 studies were included in the final meta-analysis and the effect of device wavelength, diurnal variation, imaging modality, region of interest, systemic vascular-related disease, age, axial length, refractive error, and IOP assessed in sub-analyses. The narrow prediction value and GRADE assessment further indicated that the synthesised data was robust. To the authors knowledge, at present CVI is not available as a metric on any commercial OCT software interfaces and requires external software/platforms for its calculation, limiting its use in clinical practice. However, the normative values of this review (alongside evidence that CVI is a robust biomarker of choroidal health against numerous physiological parameters) should help towards its potential integration into commercial platforms in the future.

## Conclusion

This review found average subfoveal CVI for healthy normal eyes is 66.50% (64.85–68.15%) and is independent of age, IOP and axial length when these factors are within a normative range. CVI was also independent of diurnal variation, excitation wavelength of the device, region of interest and the imaging mode used. Based on the findings from studies forming this review, we have a moderate certainty in GRADE assessment, good confidence interval, low publication bias and low to moderate heterogeneity. These findings highlight that despite the methodological differences in CVI quantification, the metric is resistant to most person, eye and image-level factors and therefore a bust measure of in vivo choroidal angioarchitecture.

## Methods

This systematic review adhered to the guidelines of the preferred reporting items for systemic reviews and meta analyses statement (PRISMA)^153,154^ and was prospectively registered on Prospero^155^ (CRD42022377271) without amendments.

### Eligibility criteria and literature search

The inclusion criteria were studies which presented the CVI of at least one, adult participant group with normal ocular health which was defined as:

- Age above 18 years,
- Absence of any ocular disease of the posterior pole (including but not limited to optic nerve head),
- Refractive error (RxE) less than ± 6 D spherical equivalent and/or axial length between or equal to 22–26 mm and,
- Intraocular pressure less than ± 21 mmHg.

Studies were included only if they calculated CVI based on methodology of Agrawal et al.^151^ which is considered the common protocol of the field. This was determined by either citation of the method or description commensurate with Agrawal et al. including marking the choroid as a region of interest of an OCT B scan, and binarizing the image using Niblack’s auto thresholding method then extracting CVI as ratio of dark pixels (Luminal Area) to total pixels (Total choroidal area). Exclusion criteria included:

- Paediatric populations,
- Calculation of CVI using alternative methodology^150^ (e.g. Sonoda et al.) or
- Studies with unclear/inadequate descriptions of the study population to determine if they met the inclusion criteria.

The presence of systemic vascular diseases was documented but not used as a criterion for exclusion.

The research question was framed using the PICO framework^156^ and the key search terms were determined prior to the literature search. These keywords were searched on electronic databases of PubMed and Embase for all published articles from inception to September 2022 to ensure adequate coverage of the literature. Given the relative novelty of this research area, search with MeSH terms was inconclusive. Instead, alternate terms for choroidal vascularity index were incorporated with Boolean operator in the text field. All subheadings were included in the search. Published journals were limited to English journals. Review articles and conference proceedings were excluded due to data limitations and initial search was compiled in Microsoft excel version 2402 (Microsoft Corporation, USA) reported in Supplementary Table 6.

### Study selection

Two independent reviewers (MK and LNS) screened all unique studies for title/abstract. Duplicates and ineligible studies were manually removed, and full text articles of potentially eligible studies were uploaded onto the Covidence website (Covidence systematic review software, Veritas Health Innovation, Melbourne, Australia. Available at www.covidence.org) for independent full text screening. Disagreements in any stages were resolved through discussion and consensus.

### Data synthesis

Data synthesis was performed by a single grader (MK) from the selected studies in Covidence. Quantitative data collected included the primary outcome measure of CVI expressed as a percentage or ratio from the control/non-intervention arm of the study. Person-level factors including age, and presence of systemic disease; image-level factors including OCT device model and scanning wavelength, imaging mode used, time of day scan was taken, and diameter of region of interest that CVI was calculated and eye-level factors including axial length, refractive error, best corrected visual acuity and intraocular pressure and was also extracted from all studies. (Detailed list of factors in Supplementary Table 7).

### Data items and quality assessment

Extracted data fields included publication details such as author list, country of publication, publication date, study design, funding sources, and conflicts of interest. The risk of bias assessment was performed using a modified version of Newcastle–Ottawa Scale (NOS) for Assessing the Quality of Non-Randomized studies in Meta-Analyses^157^ by 2 authors (MK and LNS) independently within Covidence (Supplementary Table 8).

### Statistical analysis

All the statistical analysis of the data was performed using STATA 18.0 (StataCorp LLC, Texas, USA). Since CVI can be reported as raw measure (ratio) or converted to a percentage, all the reported ratio measures of CVI were converted into percentage for homogeneity. Primary metanalysis was performed by calculation of pooled weighted average (weighted by standard error) using the inverse variance method and the random effects REML model was chosen to provide distribution of true effect sizes due to heterogeneity between studies throughout the analysis^158^. The heterogeneity was evaluated using the prediction interval rather than I^2^ statistics, to provide a more robust measure in pooled mean outcomes^159^. To assess the influence of personal factors and image based factors, sub group analysis and meta regression were performed respectively if more than 10 studies had data availability and their results were represented with forest plots. Publication bias was assessed using a Galbraith plot and confirmed with Egger regression and Begg and Mazumder rank correlation. Sensitivity analysis was performed to validate the findings from this study by

- Including and excluding studies with repeated populations, and
- Including and excluding studies with both eyes included in the sample size

Repeated populations were referred to as studies which used the same sample population as indicated by their description of similar sample size and location or explicitly stating of recruitment from specific registry or population cohort. A *p* value of < 0.05 was considered statistically significant. The overall quality assessments were expressed as percentage of low/high risk of bias. The summary of evidence was validated through GRADE assessment^160,161^.

## Electronic supplementary material

Below is the link to the electronic supplementary material.

Supplementary Material 1

## Data Availability

The dataset generated during and/or analysed during the current study are available from the corresponding author on reasonable request.
