# Supplementary material for: Choroidal vascularity index is independent of ocular and image-based factors in healthy eyes: a systematic review and meta-analysis
Source: Sci Rep. 2025 Jul 30;15:27782. doi: 10.1038/s41598-025-10384-5 (PMC12311116; doi:10.1038/s41598-025-10384-5)
Supplement: Supplementary file 1 — Supplementary Material 1 [file 41598_2025_10384_MOESM1_ESM.docx]

# Supplementary Material

**Supplementary Table 1.** List of studies (n = 99) excluded after being assessed by full text, with primary reason.

| **Study ID** | **Primary reason** | **Specific reason** |
| --- | --- | --- |
| #4-Ratra D 2018 | Paediatric population | Age <18 |
| #5-Ağın A 2019 | Paediatric population | Age <18 |
| ­#8-Murro V 2021 | Paediatric population | Age <18 |
| #10-Sun G 2021 | Wrong outcomes | Volumetric CVI |
| #11-Aslam TM 2022 | Different method | Different method of CVI extraction |
| #12-Marta A 2022 | Inclusion not clear | Inclusion criteria not met |
| #14-Borrelli 2022 | Different method | Sonada method |
| #15-Chen S 2022 | Wrong outcomes | not CVI |
| #16-Halouani S 2023 | Different method | Sonada et al method |
| #17-Abdolrahimzadeh 2022 | Different method | Sonada et al method |
| #19-Agarwal 2022 | Inclusion not clear | Inclusion criteria not met |
| #23 - Agrawal 2016 | Different method | Sonada et al method |
| #24-Agrawal 2016 | Inclusion not clear | Inclusion criteria not met |
| #25-Agrawal 2017 | Inclusion not clear | Inclusion criteria not met |
| #26 - Aksoy 2020 | Paediatric population | Age <18 |
| #27 - Aksoy 2021 | Paediatric population | Age <18 |
| #33-Atas 2022 | Inclusion not clear | Inclusion criteria not met |
| #40-Bernabei 2021 | Wrong patient population | Fellow eye as control |
| #41-Borrelli 2021 | Different method | Sonada et al method |
| #43-Breher 2020 | Different method | Sonada et al method |
| #44-Carnevali 2021 | Inclusion not clear | Inclusion criteria not met |
| #48-Chen 2022 | Wrong outcomes | Different layer |
| #49-Cheng 2022 | Wrong patient population | Individual layer CVI |
| #50-Cheng 2022 | Wrong outcomes | Not macular CVI |
| #57-Demirel 2020 | Inclusion not clear | Inclusion criteria not met |
| #58-Demirel 2022 | Different method | Sonada et al method |
| #59-Dmuchowska 2021 | Different method | Not macular CVI |
| #60-Du 2022 | Wrong outcomes | Not macular CVI |
| #61-Du 2021 | Wrong outcomes | Not macular CVI |
| #63-Faghihi 2021 | Different method | Sonada et al method |
| #64-Foo 2020 | Wrong outcomes | Not macular CVI |
| #65-Furundaoturan 2022 | Paediatric population | Age <18 |
| #66-Gediz 2021 | Different method | Different definition of CVI |
| #67 - Gediz 2022 | Paediatric population | Age <18 |
| #68-Goud 2021 | Different method | Not macular CVI |
| #69-Goud 2019 | Wrong outcomes | Not macular CVI |
| #70-Guduru 2019 | Different method | Different method |
| #72-GulerAlis 2021 | Paediatric population | Age <18 |
| #73-Gupta 2018 | Inclusion not clear | Inclusion criteria not met |
| #74-Gutierrez-Bonet 2020 | Different method | Different definition of CVI |
| #76-Hui 2022 | Paediatric population | Age <18 |
| #79-Invernizzi 2018 | Inclusion not clear | Inclusion criteria not met |
| #83-Jayakumar 2021 | Wrong patient population | Fellow eye as control |
| #84-Jiang 2021 | Wrong outcomes | Not macular CVI |
| #86-Karslioglu 2021 | Inclusion not clear | Inclusion criteria not met |
| #87-Keenan 2020 | Inclusion not clear | Inclusion criteria not met |
| #88-Kesim 2021 | Inclusion not clear | Inclusion criteria not met |
| #89 - Kim 2021 | Wrong patient population | Fellow eye as control |
| #92-Kim 2019 | Inclusion not clear | Inclusion criteria not met |
| #93-Kim 2022 | Inclusion not clear | Inclusion criteria not met |
| #94-Kim 2021 | Inclusion not clear | Inclusion criteria not met |
| #95-Kim 2022 | Wrong patient population | High myopia |
| #99-Kocamis 2022 | Inclusion not clear | Inclusion criteria not met |
| #100-Koh 2017 | Inclusion not clear | Inclusion criteria not met |
| #102-Kukan 2022 | Different method | Different definition of CVI |
| #103-Kung 2022 | Wrong outcomes | Volumetric CVI |
| #104-Lee 2020 | Inclusion not clear | Inclusion criteria not met |
| #105-Lewin 2022 | Wrong patient population | Fellow eye as control |
| #106-Li 2019 | Inclusion not clear | Inclusion criteria not met |
| #107-Liu 2019 | Different method | Sonada et al method |
| #108-Liu 2022 | Wrong patient population | High myopia |
| #111-Lu 2022 | Different method | Sonada et al method |
| #112-Lu 2022 | Inclusion not clear | Inclusion criteria not met |
| #116-Marques 2022 | Inclusion not clear | Inclusion criteria not met |
| #118-Mirzania 2022 | Inclusion not clear | Inclusion criteria not met |
| #119-Mori 2022 | Inclusion not clear | Inclusion criteria not met |
| #122-Oh 2020 | Paediatric population | Age <18 |
| #123-Okamoto 2018 | Different method | Sonada et al method |
| #126-Ozcan 2022 | Wrong outcomes | Not macular CVI |
| #127 - OzcelikKose 2022 | Inclusion not clear | Inclusion criteria not met |
| #130-OzdamarErol 2021 | Wrong patient population | Fellow eye as control |
| #132-Ozsaygili 2021 | Different method | Methodology unclear |
| #136-Polat 2022 | Wrong outcome | not CVI |
| #137-Qian 2021 | Different method | Different measure |
| #138-Rishi 2022 | Paediatric population | Age <18 |
| #142-Sacconi 2021 | Different method | Sonada et al method |
| #145-SekeryapanGediz 2022 | Different method | Different definition of CVI |
| #146-Seo 2022 | Inclusion not clear | Inclusion criteria not met |
| #148-Shen 2020 | Wrong outcomes | Volumetric CVI |
| #149-Shin 2019 | Wrong patient population | No control group |
| #154 Singh 2018 | Different method | Sonada et al method |
| #155 Singh 2019 | Different method | Sonada et al method |
| #156-Singh 2021 | Different method | Sonada et al method |
| #158-Suzuki 2021 | Inclusion not clear | Inclusion criteria not met |
| #166-Temel 2021 | Inclusion not clear | Inclusion criteria not met |
| #169-Toto 2022 | Inclusion not clear | Inclusion criteria not met |
| #171-Ugurlu 2022 | Wrong outcomes | Not macular CVI |
| #174-Vupparaboina 2018 | Wrong outcomes | Not macular CVI |
| #178-Wei 2019 | Inclusion not clear | Inclusion criteria not met |
| #183-Xu 2021 | Wrong outcomes | Not macular CVI |
| #184-Yan 2022 | Wrong patient population | Fellow eye as control |
| #185-Yang 2020 | Wrong outcomes | Not macular CVI |
| #187-Yanik 2022 | Inclusion not clear | Inclusion criteria not met |
| #194-Yoon 2022 | Inclusion not clear | Inclusion criteria not met |
| #195-Zeng 2022 | Wrong outcomes | Volumetric CVI |
| #196-Zhang 2022 | Wrong outcomes | Different layer |
| #197-Zhou 2020 | Wrong outcomes | Not macular CVI |
| #198-Zhu 2022 | Paediatric population | Age <18 |
| #199-Guduru 2020 | Paediatric population | Age <18 |

**Supplementary Table 2. Data extracted from the included studies**

Abbreviations: CVI – choroidal vascularity index,

| **#ID Study** | **Mean Age** | **Sample Size** | **Region of interest (in diameter)** | **OCT Device** | | | **BCVA** | **Testing conditions** | | | **Mean CVI** |
| --- | --- | --- | --- | --- | --- | --- | --- | --- | --- | --- | --- |
|  |  |  |  | **Device used** | **Wavelength** | **Mode of imaging** |  | **Refractive error** | **Time of scan** | **Systemic diseases** |  |
| #18-Agarwal 2018 | 33.6 | 30 | >3 mm | Spectralis OCT | 870 | EDI Mode | 0.01 | Not reported | 10.00 am to 12.00 pm | Not reported | 66.9 |
| #20-Agarwal 2020 | 33.6 | 30 | >3 mm | SSOCTA A DRI Triton, Topcon | 1050 | Standard | Not reported | Not reported | Not reported | Absent | 66.00 |
| #21-Agrawal 2016 | 61.53 | 345 | 1.5 mm | Spectralis OCT | 870 | EDI Mode | Not reported | Not reported | Not reported | Not reported | 65.61 |
| #1-Agrawal 2016 | 37.4 | 16 | >3 mm | Spectralis OCT | 870 | EDI Mode | 0.00 | Not reported | Not reported | Not reported | 65.18 |
| #22-Agrawal 2021 | 29.2 | 6 | >3 mm | SS OCT | 1050 | Standard | Not reported | -0.3 | 10.00-11.00 am | Not reported | 56.79 |
| #28-Aksoy 2022 | 39.3 | 50 | 1.5 mm | Spectralis OCT | 870 | EDI Mode | 0.00 | Not reported | 9.00 am -11.00am | Absent | 66.62 |
| #29-Alis 2022 | 58.3 | 40 | >3 mm | EDI OCT | 670 | EDI Mode | Not reported | Not reported | 8 Am to 2.00PM | Present | 68 |
| #30-Altinel 2022 | 48.65 | 26 | 1.5 mm | Cirrus EDI OCT | 840 | EDI Mode | Not reported | Not reported | 9.00 am to 12.00 pm | Not reported | 69.22 |
| #32-Asikgarip 2021 | 25.1 | 36 | 3000mm | Spectralis OCT | 870 | EDI Mode | 0.00 | Not reported | 9.00am -12.00 pm | Absent | 71.04 |
| #31-Asikgarip 2022 | 50.9 | 50 | >3 mm | EDI OCT | 670 | EDI Mode | Not reported | Not reported | Not reported | Absent | 74.83 |
| #34-Azuma 2021 | 35.5 | 13 | 1.5 mm | Spectralis OCT | 870 | EDI Mode | Not reported | -2.3 | Not reported | Absent | 60.8 |
| #35-Bakthavatsalam 2017 | 65.1 | 72 | 1.5 mm | SS OCT DRI Triton | 1050 | Standard | 0.3 | Not reported | 9.00 am to 12.00 am | Absent | 68.53 |
| #38-Balci 2020 | 42.85 | 20 | 1.5 mm | Spectralis OCT | 870 | EDI Mode | Not reported | 0.4 | 9.00 am to 11.00am | Not reported | 61.1 |
| #37-Balci 2021 | 33.4 | 30 | >3 mm | Spectralis OCT | 870 | EDI Mode | 0.00 | -0.3 | 9.00am to 11.00 am | Not reported | 63.9 |
| #36-Balci 2022 | 40.17 | 42 | 1.5 mm | Spectralis OCT | 870 | EDI Mode | 0.00 | Not reported | 5.30(am/pm not mentioned) and 7.30 pm | Absent | 71 |
| #39-Bayram 2022 | Not reported | 53 | >3 mm | Spectralis OCT | 870 | Standard | Not reported | Not reported | Not reported | Not reported | 64.00 |
| #42-Bousquet 2021 | 60 | 22 | 1.5 mm | Spectralis OCT | 870 | EDI Mode | 0.00 | 0.1 | Not reported | Not reported | 67.5 |
| #45-Ceri 2022 | 45.8 | 30 | >3 mm | Spectralis OCT | 870 | EDI Mode | Not reported | 0.1 | 10 am to 12 pm | Absent | 65.00 |
| #46-Cevher 2022 | 25.33 | 33 | 1500 microns, 7500 microns | Spectralis OCT | 870 | EDI Mode | 0.00 | -0.5 | 9.00 and 11 AM | Not reported | 65.00 |
| #47-Ceylanoglu 2022 | 42.2 | 64 | >3 mm | Spectralis OCT | 870 | EDI Mode | Not reported | Not reported | Not reported | Absent | 57 |
| #51-Cheong 2022 | 53.8 | 50 | 1.5 mm | Spectralis OCT | 870 | EDI Mode | Not reported | -0.7 | Not reported | Present | 63.53 |
| #52-Chun 2022 | 65.47 | 30 | 1.5 mm | SS OCT DRI Triton | 1050 | Standard | 0.04 | -0.1 | Not reported | Not reported | 63.33 |
| #53-Cicinelli 2022 | 40.3 | 18 | 1.5 mm | OCTA plex elite and Spectralis OCT | 840 | Standard | 0.00 | -2.4 | Not reported | Absent | 65.6 |
| #54-Damian 2021 | 53.28 | 36 | 1.5 mm | Spectralis OCT | 870 | EDI Mode | 0.02 | Not reported | 1:00 pm to 3.00 pm | Absent | 74.14 |
| #55-Dave 2022 | 42.75 | 18 | 1.5 mm | SS OCT Topcon DRI OCT | 1050 | Standard | 0.00 | 0.3 | 2:00 p.m. and 3:00 p.m | Absent | 63.95 |
| #56-DeBernardo 2021 | 40.67 | 67 | >3 mm | Spectralis OCT | 870 | EDI Mode | Not reported |  | 8.00 am to 11.00 am | Absent | 67.55 |
| #62-Durusoy 2021 | 33.81 | 62 | >3 mm | Optovue RTVue XR SD OCT | 840 | Standard | 0.00 | 0.5 | Not reported | Not reported | 68.3 |
| #6-G Giannaccare  2020 | 73.9 | 32 | >3 mm | Spectralis OCT | 870 | EDI Mode | 0.04 | Not reported | Not reported | Not reported | 69.33 |
| #13-G LEDESMA-GIL 2022 | 31 | 10 | >3 mm | SS OCT  DRI-OCT Triton OCT | 1050 | Standard | Not reported | Not reported | Not reported | Not reported | 71.81 |
| #71-GulerAlis 2021 | Not reported | 100 | 3.0mm | RS-3000 Advance OCT | 880 | EDI Mode | Not reported | -0.05 | Not reported | Not reported | 68 |
| #75-Hepokur 2021 | 28 | 17 | >3 mm | Spectralis OCT and OCTA | 870 | EDI Mode | 1.00 | Not reported | Not reported | Absent | 75.00 |
| #77-Hwang 2022 | Not reported | 20 | 1.5 mm | SS OCT(Topcon DRI OCT  Triton | 1050 | Standard | Not reported | Not reported | Not reported | Absent | 63.46 |
| #78-Inam 2019 | Not reported | 19 | >3 mm | Spectralis OCT | 870 | EDI Mode | Not reported | Not reported | 10.00 am to 12.00 am ( am stated in the paper/ probably meant pm) | Not reported | 65.66 |
| #80-Iovino 2021 | 52.3 | 34 | >3 mm | Spectralis OCT | 870 | Standard | Not reported | -0.6 | 10.am to 12 am | Not reported | 65.58 |
| #81-Iovino 2022 | 35.1 | 69 | >3 mm | Spectralis OCT | 870 | Standard | Not reported | Not reported | not specified, only mentioned that it was performed at the same time of the day | Not reported | 64.00 |
| #82-Isik 2022 | 35.9 | 50 | >3 mm | Cirrus HD OCT-5000 | 840 | EDI Mode | Not reported | Not reported | 9.30am to 2.00 pm | Absent | 64.3 |
| #85-Karasu 2021 | 58.34 | 38 | 1.5 mm | SS OCT DRI-OCT Triton OCT Topcon | 1050 | Standard | Not reported | Not reported | Not reported | Absent | 68.00 |
| #91-Kim 2018 | 57.47 | 45 | 1.5 mm | SS OCT DRI-OCT Triton OCT Topcon | 1050 | Standard | 0.07 | -0.7 | Not reported | Absent | 69.08 |
| #90-Kim 2018 | 51.7 | 42 | >3 mm | SS OCT and OCT  DRI-OCT Triton OCT Topcon | 1050 | Standard | 0.06 | -0.9 | 9.00 am to 12.00 pm | Absent | 69.21 |
| #98-Kocak 2020 | 41.82 | 46 | 1.5 mm | Spectralis OCT | 870 | EDI Mode | 0.00 | -0.3 | 9.00 am to 12.00 pm | Absent | 66.3 |
| #97-Kocak 2021 | Not reported | 224 | 1.5 mm | Spectralis OCT | 870 | EDI Mode | Not reported | Not reported | 10.00am to 12.00 pm |  | 66.9 |
| #96-Kocak 2022 | 31.38 | 28 | >3 mm | Spectralis OCT | 870 | EDI Mode | Not reported | -0.6 | Not reported | Absent | 67.67 |
| #101-Krytkowska 2021 | 73.1 | 121 | 1.5 mm | Spectralis OCT | 870 | EDI Mode | Not reported | Not reported | 9 am to 1 pm | Present | 66.00 |
| #109-Liu 2018 | 37.3 | 40 | >3 mm | Spectrallis OCT | 870 | EDI Mode | Not reported | -0.8 | 12.00 pm to 14.00 pm | Not reported | 70.00 |
| #110-Loiudice 2021 | 37.45 | 40 | >3 mm | Spectralis OCT | 870 | EDI Mode | Not reported | Not reported | Not reported | Absent | 64.78 |
| #114-Ma 2022 | 71 | 120 | 2mm | Cirrus HD-OCT 5000, standard, Zeiss Cirrus  HD-OCT 5000 | 840 | Standard | Not reported | Not reported | Not reported | Not reported | 68.00 |
| #113-Ma 2022 | 48.65 | 80 | >3 mm | Cirrus HD-OCT 5000 , OCTA | 840 | Standard | Not reported | Not reported | Not reported | Not reported | 63.99 |
| #115-Magesan 2022 | 34.83 | 26 | >3 mm | SSOCTA, PLEX Elite 9000 device | 1050 | EDI Mode | Not reported | 0.5 | Not reported | Not reported | 66.55 |
| #9-MG Ersoz  2021 | 30.9 | 32 | >3 mm | Spectralis OCT | 870 | Standard | Not reported | 2.4 | 3pm to 6pm | Not reported | 66 |
| #117-Mirshahi 2022 | 42.38 | 74 | 1.5 mm | Spectralis OCT | 870 | EDI Mode | Not reported | Not reported | Not reported | Not reported | 71.86 |
| #120-Nivison-Smith 2020 | 48.27 | 106 | 1.5 mm | Spectralis OCT | 870 | Standard | 0.04 | -2.3 | 1.00 to 3.00pm | Absent | 68.09 |
| #121-Obada 2022 | 71.4 | 21 | 1.5 mm | SS OCT, DRI Triton SS-OCT | 1050 | Standard | 0.26 | 0.3 | Not reported | Present | 66.21 |
| #124-Ozcaliskan 2020 | 71.7 | 60 | 3.00mm | Spectralis OCT | 870 | Standard | Not reported | -0.6 | 1.00 pm to 3.00 pm | Not reported | 62.7 |
| #125-Ozcan 2021 | 56.3 | 40 | 1.5 mm | Spectralis OCT | 870 | EDI Mode | Not reported | Not reported | 9.00 am to 11.00 am | Not reported | 73.39 |
| #128-Ozcelik-Kose 2021 | 42.8 | 30 | 1.5 mm | Spectralis OCT | 870 | EDI Mode | Not reported | -2.4 | Not reported | Not reported | 61.1 |
| #129-Ozcelik-Kose 2022 | 27.35 | 25 | >3 mm | Spectralis OCT | 870 | EDI Mode | Not reported | -0.01 | 9.00 am to 10.00 am | Absent | 69.29 |
| #131-Ozer 2020 | 32.4 | 24 | central 1 mm on ETDRS grid | Spectralis OCT | 870 | EDI Mode | 0.00 | Not reported | Not reported | Not reported | 69.00 |
| #133-Parisi 2020 | 70.02 | 20 | 1.5mm | Spectralis OCT | 870 | EDI Mode | Not reported | Not reported | Not reported | Not reported | 66.00 |
| #134-Park 2019 | 67.3 | 50 | >3 mm | SS OCT DRI-OCT system | 1050 | Standard | 0.13 | Not reported | 9:00 AM | Absent | 68.81 |
| #135-Pellegrini 2019 | 77.2 | 20 | >3 mm | Spectralis OCT | 870 | EDI Mode | 0.04 | Not reported | Not reported | Not reported | 70 |
| #140-Robbins 2021 | 70.9 | 248 | >3 mm | Cirrus HD-5000 SD OCT, angioplex | 840 | EDI Mode | Not reported | Not reported | Not reported | Not reported | 63.53 |
| #139-Robbins 2021 | 69.23 | 248 | >3 mm | Cirrus HD-5000 SD OCT | 840 | EDI Mode | 0.099 | Not reported | Not reported | Not reported | 63.8 |
| #141-Sacconi 2022 | 71.3 | 18 | >3 mm | Spectralis OCT | 870 | EDI Mode | 0.05 | Not reported | Not reported | Not reported | 60.35 |
| #7-SB Velaga 2020 | 72.33 | 17 | >3 mm | Spectralis OCT | 870 | EDI Mode | Not reported | Not reported | Not reported | Not reported | 53.43 |
| #143-Scarinci 2021 | 50 | 17 | 1.5mm | Spectralis OCT | 870 | EDI Mode | 87.1 | Not reported | 10 am to 12 pm | Not reported | 62.26 |
| #144-SekeryapanGediz 2021 | 24.1 | 23 | 1.5mm | Spectralis OCTA | 870 | EDI Mode | 0.00 | Not reported | 12.00 pm to 4.00 pm | Not reported | 62.8 |
| #147-Sevik 2022 | 31.1 | 60 | >3 mm | Spectral OCT | 870 | EDI Mode | 0.00 | Not reported | Not reported | Absent | 62.19 |
| #150-Shrivastav 2022 | 40.9 | 61 | 1 mm | Cirrus 5000 Carl Zeiss SD OCT | 840 | EDI Mode | Not reported | Not reported | 8.00 am to 11.00 am | Not reported | 64 |
| #151-Sidorczuk 2022 | 55.73 | 76 | 1.5mm | Spectralis OCT | 870 | Standard | Not reported | 0.4 | 9.00 am to 11.00 am | Not reported | 65.00 |
| #153-Simsek 2022 | 57.1 | 48 | >3 mm | Spectralis OCT | 870 | Standard | 0.00 | -0.8 | 9.00 and 11.30 am | Not reported | 69.3 |
| #152-Simsek 2022 | 33.5 | 35 | Wide field and 2 discs to fovea image cropped | Spectralis OCT | 870 | EDI Mode | Not reported | -0.9 | 9.00am to 12.00pm | Not reported | 70.64 |
| #157-Su 2020 | 30.08 | 23 | 1.5 | Spectralis OCT | 870 | EDI Mode | Not reported | Not reported | Not reported | Absent | 67.31 |
| #159-Tan 2016 | 70 | 38 | >3 mm | OCT-1000 Topcon Corp SD OCT EDI mode | 840 | EDI Mode | Not reported | Not reported | Not reported | Not reported | 67.2 |
| #160-Tan 2018 | 40.04 | 26 | 3 mm | SS OCT DRI-OCT Triton | 1050 | Standard | 0.02 | Not reported | 9.00 am to 12.00pm | Not reported | 59.47 |
| #165-Temel 2021 | 29 | 25 | 3.00mm | Spectralis OCT | 870 | EDI Mode | Not reported | Not reported | 9.00 am to 12.00 pm | Not reported | 66.8 |
| #164-Temel 2021 | 51.1 | 48 | 3.00mm | Spectralis OCT | 870 | EDI Mode | Not reported | Not reported | 1.00 pm to 3.00 pm | Absent | 74.93 |
| #162-Temel 2021 | 38.9 | 28 | 3mm | Spectralis OCT | 870 | Standard | 0.00 | -0.8 | 9.00 am to 12.00pm | Absent | 73.38 |
| #167-Temel 2022 | 49.8 | 30 | 1.5mm | Spectralis OCT | 870 | EDI Mode | Not reported | -0.4 | 9.00 am to 12.00 pm | Not reported | 73.52 |
| #163-Temel 2022 | 34.5 | 36 | 1.5mm | Spectralis OCT | 870 | EDI Mode | Not reported | -0.5 | Not reported | Not reported | 74.8 |
| #161-Temel 2022 | 36.5 | 40 | 3.00mm | Spectralis OCT | 870 | EDI Mode | 0.00 | -0.8 | 9.00am to 12.00 pm | Absent | 73.41 |
| #168-Topcuoglu 2021 | 49.6 | 26 | 1.5mm | (RTVue-XR Avanti, Optovue Inc., | 840 | Standard | 0.12 | Not reported | 8.30 am to 11.00 am | Absent | 64.00 |
| #170-UcgulAtilgan 2023 | 76.95 | 47 | >3 mm | Spectralis OCT | 870 | EDI Mode | 0.07 | 0.3 | 8.00 am to 2.00 pm | Absent | 64.81 |
| #172-Uslu 2021 | 47.03 | 38 | >3 mm | Cirrus HD-OCT 4000 ,SD OCT, | 840 | EDI Mode | Not reported | Not reported | 9.00 am to 11.00 am | Absent | 69.21 |
| #173-Viggiano 2022 | 76.06 | 30 | >3 mm | Spectralis OCT | 870 | Standard | Not reported | Not reported | Not reported | Absent | 72.00 |
| #177-Wang 2021 | 59.83 | 203 | 1.5mm | HD-OCT | 840 | Standard | Not reported | -0.9 | Not reported | Absent | 66.90 |
| #175-Wang 2021 | 26.58 | 78 | >3 mm | Cirrus HD-OC, SD OCT | 840 | EDI Mode | -0.27 | -4.26 | Not reported | Not reported | 63.60 |
| #176-Wang 2022 | 38 | 42 | >3 mm | SS-OCT, VG200S; SVision Imaging | 1050 | Standard | -0.1 | 0.9 | Not reported | Not reported | 61 |
| #181-Wei 2018 | 38.13 | 100 | >3 mm | Spectralis OCT | 870 | EDI Mode | 0.00 | -0.1 | Not reported | Not reported | 70.12 |
| #179-Wei 2019 | 32 | 32 | >3 mm | Spectralis OCT | 870 | EDI Mode | Not reported | Not reported | Not reported | Not reported | 69.5 |
| #180-Wei 2022 | 36.71 | 242 | >3 mm | Spectralis OCT | 870 | EDI Mode | Not reported | Not reported | 1.30 pm to 5.00 pm | Not reported | 67.50 |
| #182-Wu 2021 | 23.4 | 34 | >3 mm | SS-OCT  VG200S; SVision Imaging | 1050 | Standard | 0.00 | 1.3 | 2.00 pm to 5.00 pm | Absent | 61 |
| #186-Yang 2022 | 22.04 | 130 | >3 mm | Cirrus HD-OCT ,SD OCT | 840 | EDI Mode | -0.08 | -4.7 | Not reported | Absent | 63.09 |
| #188-Yao 2020 | 67 | 23 | >3 mm | SS OCT Triton DRI-OCT | 1050 | Standard | Not reported | Not reported | 1.00 pm to 6.00 pm | Absent | 62.77 |
| #189-Yazdani 2021 | 30 | 29 | 1.5mm | Spectralis OCT | 870 | Standard | Not reported | Not reported | 9.00 am to 12.00 pm | Absent | 54.4 |
| #190-Yeter 2021 | 46.9 | 53 | >3 mm | Spectralis OCT | 870 | EDI Mode | Not reported | -0.6 | 8.00 am baseline and 12.00 pm post mask | Absent | 67 |
| #191-Yildiz 2021 | 26 | 20 | 1.5mm | Spectralis OCT | 870 | EDI Mode | Not reported | -0.5 | 9.00 am to 11.00 am | Absent | 66 |
| #192-Yilmaz 2021 | 44.3 | 50 | 1.5mm | Spectralis OCT | 870 | EDI Mode | 0.00 | -1.0 | Not reported | Absent | 69.5 |
| #193-Yip 2019 | 68.9 | 18 | 1.5mm | Spectralis OCT | 870 | EDI Mode | Not reported | -1.6 | Not reported | Absent | 67.3 |

**Supplementary Table 3.** Funding and conflict of interest statements (*verbatim*).

| **Study** | **Funding** | **Conflict of interest** |
| --- | --- | --- |
| #18-Agarwal 2018 | Department of Biotechnology India for the development of Center of Excellence at the Advanced Eye Center, PGIMER Chandigarh, India | The authors declare no competing interests |
| #20-Agarwal 2020 | Not funded | The authors declare no competing interests |
| #21-Agrawal 2016 | National Medical Research Council, Singapore [Grant No. NMRC R760/44/2010]. | The authors declare no competing interests |
| #1-Agrawal 2016 | Not funded | The authors declare no competing interests |
| #22-Agrawal 2021 | Not funded | The authors declare no competing interests |
| #28-Aksoy 2022 | BaÅŸkent University Research Fund | The authors declare no competing interests |
| #29-Alis 2022 | Not funded | The authors declare no competing interests |
| #30-Altinel 2022 | Not funded | The authors declare no competing interests |
| #32-Asikgarip 2021 | Not funded | The authors declare no competing interests |
| #31-Asikgarip 2022 | Not funded | The authors declare no competing interests |
| #34-Azuma 2021 | Not funded | The authors declare no competing interests |
| #35-Bakthavatsalam 2017 | Not funded | The authors declare no competing interests |
| #38-Balci 2020 | Not funded | The authors declare no competing interests |
| #37-Balci 2021 | Not funded | The authors declare no competing interests |
| #36-Balci 2022 | Not funded | The authors declare no competing interests |
| #39-Bayram 2022 | Not funded | The authors declare no competing interests |
| #42-Bousquet 2021 | Not funded | The authors declare no competing interests |
| #45-Ceri 2022 | Not funded | The authors declare no competing interests |
| #46-Cevher 2022 | Not funded | The authors declare no competing interests |
| #47-Ceylanoglu 2022 | Not funded | The authors declare no competing interests |
| #51-Cheong 2022 | Duke/Duke-NUS Research Collaborations Grant: Duke/Duke-NUS/RECA(Pilot)2016/0020, Biomedical Research Council Singapore Grant: SPF2014/002, National Medical Research Council Open Fund Large Col-laborative Grant: NMRC/LCG/004/2018, NMRC/CG-INCEPTOR/Pre-Clinical Core Platform/2017_SERI, and the National Medical Research Council Clinician Scientist Individual Research Grants: NMRC/CIRG/1417/2015, and NMRC/CIRG/1488/2018 | The authors declare no competing interests |
| #52-Chun 2022 | National Research Foundation of Korea (NRF- 2020R1F1A1074898). | The authors declare no competing interests |
| #53-Cicinelli 2022 | Not funded | The authors declare no competing interests |
| #54-Damian 2021 | Not funded | The authors declare no competing interests |
| #55-Dave 2022 | This work was supported by the Hyderabad Eye Research | The authors declare no competing interests |
| #56-DeBernardo 2021 | Not funded | The authors declare no competing interests |
| #62-Durusoy 2021 | Not funded | The authors declare no competing interests |
| #6-G Giannaccare  2020 | Not funded | The authors declare no competing interests |
| #13-G LEDESMA-GIL 2022 | The Macula Foundation, Inc., New York, NY | R.F. Spaide has received personal compensation from Topcon Medical Systems, Roche, Genentech, Bayer, Regeneron, Heidelberg Engineering, Adverum Biotechnologies, and DORC. Z. Maoand Jonathan Liu works for Topcon Medical Systems at Topcon Advanced Biomedical Laboratory. The remaining authors have any financial/conflicting interests to disclose. |
| #71-GulerAlis 2021 | Not funded | The authors declare no competing interests |
| #75-Hepokur 2021 | Not funded | The authors declare no competing interests |
| #77-Hwang 2022 | This study was supported by the Basic Science Research  Program through the National Research Foundation of Korea (NRF-2020R1F1A1074898). | The authors declare no competing interests |
| #78-Inam 2019 | Not funded | The authors declare no competing interests |
| #80-Iovino 2021 | Not funded | The authors declare no competing interests |
| #81-Iovino 2022 | The present study was supported by the Research Project â€œ, Toward the development of novel biomarkers to evaluate experimental therapies for Inherited Retinal Dystrophies: correlation between Magnetic Resonance Imaging and visual functionâ€ funded by Italian Ministry of Education, University and Research (Grant PRIN 2017 n 20177EJ75C). | The authors declare no competing interests |
| #82-Isik 2022 | Not funded | No conflict of interest declared by authors |
| #85-Karasu 2021 | Not funded | The authors declare no competing interests |
| #91-Kim 2018 | This research was supported by the Basic Science Research Program through the National Research Foundation of Korea (NRF), funded by the Ministry of Education (2016R1A6A1A03010528). | The authors declare that they have no competing interests |
| #90-Kim 2018 | This research was supported by the Basic Science Research Program through the National Research Foundation of Korea (NRF), funded by the Ministry of Education (2016R1A6A1A03010528). | The authors declare no competing interests |
| #98-Kocak 2020 | Not funded | The authors report no conflict of interest |
| #97-Kocak 2021 | The authors received no financial support for the research, authorship, and/or publication of this article | The authors declare that there is no conflict of interest. |
| #96-Kocak 2022 | The author(s) received no financial support for the research, authorship, and/or publication of this article. | The author(s) declared no potential conflicts of interest with respect to the research, authorship, and/or publication of this article |
| #101-Krytkowska 2021 | This work was supported by Polish National Centre for Research and Development (grant number:   STRATEGMED1/234261/2NCBR/2014) and European Union funds from the European Union Regional Development Fund, Interreg Cooperation Program V A Mecklenburg-Western Pomerania/Brandenburg/Poland for 2014â€“2020:Consolidating cross-border cooperation through ex-change of knowledge and skills in the field of modern diagnostic imaging methods in ophthalmology | The authors declare no competing interests. |
| #109-Liu 2018 | This work was supported by the Chongqing Key Laboratory of Ophthalmology (CSTC, 2008CA5003), the National Key Clinical Specialties Construction Program of China, the Key Project of Health Bureau of Chongqing (2012-1-003).The Chongqing Science & Technology Platform and Base Construction Program (cstc2014pt-sy10002), the Major Research Development Program of China (2016YFC0904000), and the Bethune Charitable Foundation (BJ-LM2016004L). The sponsor or funding organization had no role in the design or conduct of this research. | The authors report no conflicts of interest. |
| #110-Loiudice 2021 | Not funded | The authors declare that they have no competing interests |
| #114-Ma 2022 | This study was supported in part by the Alzheimer Drug Discovery Foundation and by a Duke-Duke/NUS Pilot  Collaborative Award. | None of the authors listed have a proprietary interest in the material presented or any conflicts of interest to disclose. |
| #113-Ma 2022 | This study was funded by a technology research plan from the Department of Science and Technology of Qinghai Province (grant number 2019-ZJ-7064). | The authors declare no competing interests |
| #115-Magesan 2022 | Not funded | The authors declare no competing interests |
| #9-MG Ersoz  2021 | Not funded | None |
| #117-Mirshahi 2022 | Not stated | The authors declare no competing interests |
| #120-Nivison-Smith 2020 | This work was supported, in part, by a research Grant from the Rebecca Cooper Foundation. Guide Dogs NSW/ ACT provides support for the Centre for Eye Health (the clinic of recruitment), and authors LN-S and MK. | Dr Nivison-Smith and Professor Kalloniatis have a patent named Bio-Imaging of the Eye Using Pattern Recognition (No. 2018901002). The authors declare no competing interests |
| #121-Obada 2022 | Not funded | The authors declare no conflict of interest |
| #124-Ozcaliskan 2020 | The author(s) received no financial support for the research,authorship, and/or publication of this article. | The author(s) declared no potential conflicts of interest with respect to the research, authorship, and/or publication of this article |
| #125-Ozcan 2021 | Not stated | The authors declare no competing interests |
| #128-Ozcelik-Kose 2021 | This research did not receive any specific grant from funding  agencies in the public, commercial, or not-for-profit sectors. | The authors declare no conflict of interest. |
| #129-Ozcelik-Kose 2022 | Not stated | The authors declare no competing interests |
| #131-Ozer 2020 | No commercial relationship exists for any of this article authors in the form of financial support or personal financial interest | The authors declare no competing interests |
| #133-Parisi 2020 | The contribution of IRCCS- Fondazione Bietti was supported by the Italian Ministry of Health and Fondazione Roma. The authors alone are responsible for the content and writing of the paper. | The authors declare no competing interests |
| #134-Park 2019 | This study is supported by a grant of the National Research Foundation of Korea (NRF) grant funded by the Korean government (NRF2016R1C1B2016649) to KJC. The funders had no role in study design, data collection, and analysis, decision to publish, or preparation of the manuscript. | The authors declare no competing interests |
| #135-Pellegrini 2019 | Not funded | The authors declare no competing interests |
| #140-Robbins 2021 |  | Dr Scott reported receiving grants from the Biogen clinical trial, the Vaccinex clinical trial, the CHDI Foundation clinical trial, and the Neurocrine Biosciences clinical trial outside of the submitted study. No other disclosures were reported. |
| #139-Robbins 2021 | Alzheimer Drug Discovery Foundation (D.S.G., S.F.), Karen L. Wrenn Alzheimer Grant (C.B.R. ) | The authors declare no competing interests |
| #141-Sacconi 2022 | GV, SB, and MB: none. KKV: None. JC: Allergan Inc(Irvine, California, USA), Biogen(Cambridge, Massachusetts, USA), Salutaris, OD-OS. RS is a consultant for: Novartis (Basel, Switzerland), and Zeiss (Dublin, USA). EB is a  consultant for: Novartis (Basel,Switzerland), and Zeiss (Dublin, USA). FB is a consultant for Alcon (Fort Worth,Texas,USA), Alimera Sciences (Alpharetta, Georgia, USA), Allergan Inc (Irvine, California,USA), Farmila-Thea (Clermont-Ferrand, France), Bayer Shering-Pharma (Berlin,Germany), Bausch And Lomb (Rochester, New York, USA), Genentech (San Francisco,California, USA), Hoffmann-La-Roche (Basel, Switzerland), NovagaliPharma (Ã‰vry,France), Novartis (Basel, Switzerland), Sanofi-Aventis (Paris, France), Thrombogenics (Heverlee, Belgium), Zeiss (Dublin, USA). GQ is a consultant for Alimera Sciences(Alpharetta, Georgia, USA), Allergan Inc (Irvine, California, USA), Amgen (ThousandOaks, USA), Heidelberg (Germany), KBH (Chengdu, China), LEH Pharma (London, UK),Lumithera (Poulsbo, USA), Novartis (Basel,Switzerland), Bayer Shering-Pharma (Berlin,Germany), Sandoz (Berlin, Germany), Sifi (Catania, Italy), Soof-Fidia (Albano, Italy),Zeiss (Dublin, USA) | The authors declare no competing interests |
| #7-SB Velaga 2020 | National Eye Institute, Bethesda, Maryland Grant #RO1EY023164 | S. R. Sadda is a consultant for and receives research support from Optos and Carl Zeiss Meditec, and serves as a consultant for Centervue, and has access to research instruments provided by Heidelberg Engineering, Topcon Medical Systems, Optos, CarlZeiss Meditec, Nidek, and Centervue |
| #143-Scarinci 2021 | Supported by the Italian Ministry of Health and Fondazione Roma | The authors declare no competing interests |
| #144-SekeryapanGediz 2021 | Not funded | The authors declare no competing interests |
| #147-Sevik 2022 | Not funded | The authors declare no competing interests |
| #150-Shrivastav 2022 | Not funded | The authors declare no competing interests |
| #151-Sidorczuk 2022 | This work was supported by the Medical University of Bialystok, Poland (grant no.SUB/1/DN/21/002/1157) | The authors declare no competing interests |
| #153-Simsek 2022 | The authors indicate they have no financial disclosures. | The authors declare no competing interests |
| #152-Simsek 2022 | Not funded | The authors declare no competing interests. |
| #157-Su 2020 | Supported by the NIH Funding (R21EY03029501A1) and unrestricted grant by Research to Prevent Blindness given to the Stein Eye Institute | Disclosure: L. Su, None; W. Taweebanjongsin, None; S.L. Gaw, None; G. Rabina, None; S.R. Sadda, Genentech (I), Allergan (I), Novartis (I), 4DMT (I), Oxurion (I), Optos (I, R), Heidelberg (I), Carl Zeiss Meditec (R), Centervue (I), Topcon (I), Amgen (I); I. Tsui, None |
| #159-Tan 2016 | The study was funded by Pitch for funds grant call administered by Tan Tock Seng Hospital, Singa- pore | The authors declare no competing interests |
| #160-Tan 2018 | Not funded | The authors declare no competing interests |
| #165-Temel 2021 | Not funded | The authors declare no competing interests |
| #164-Temel 2021 | Not funded | The authors declare no competing interests |
| #162-Temel 2021 | Not funded | The authors declare no competing interests |
| #167-Temel 2022 | Not funded | The authors declare no competing interests |
| #163-Temel 2022 | Not funded | The authors declare no competing interests |
| #161-Temel 2022 | Not funded | The authors declare no competing interests |
| #168-Topcuoglu 2021 | Not funded | The authors declare no competing interests |
| #170-UcgulAtilgan 2023 | Not stated | The authors declare no competing interests |
| #172-Uslu 2021 | Not funded | The authors declare no competing interests |
| #173-Viggiano 2022 | Not funded | The authors declare no competing interests |
| #177-Wang 2021 | Health and Medical Research Fund, Hong Kong (Ref. No.05162836 to C. C. T.). General Research Fund, Hong Kong (Ref. No. 14107516 to C. C. T.). The funding organization had no role in the design or conduct of this research. | The funding organization had no role in the design or conduct this research |
| #175-Wang 2021 | This study was supported by Natural Science Foundation of Beijing, China (grant number 7202229) | The authors declare no competing interests |
| #176-Wang 2022 | Supported by research grants from the Key R&D Program Projects in Zhejiang Province (2019C03045); Natural Science Foundation of Zhejiang Province (LQ21H120007); National Nature Science Foundation of China(8210041176); National Key Research and Development Program of China (2020YFC2008200); Wenzhou Municipal Science and Technology Bureau (2018ZY016); Research Fund of Wenzhou Institute, Chinese Academy of Sciences (WIUCASYJ2020004, WIUCASQD2020009); and National Nature Science Foundation of China (Grant No. 82101177). | The authors declare no competing interests |
| #181-Wei 2018 | Not stated | The authors declare no competing interests |
| #179-Wei 2019 | Not stated | The authors declare no competing interests |
| #180-Wei 2022 | No funding or sponsorship was received for this study or publication of this article. | The authors declare no competing interests |
| #182-Wu 2021 | Supported by grants from the National Natural Science Foundation of China (81830027, 81970833, 81670886, 82000931), National Key Research and Development Program of China Choroidal Blood Flow in Anisomyopes  IOVS \| January 2021 \| Vol. 62 \| No. 1 \| Article 8 \| 9 (2019YFC1710204), and CAMS Innovation Fund for Medical Sciences (2019-I2M-5-048) | The authors declare no competing interests |
| #186-Yang 2022 | This study was supported by Natural Science Foundation of Beijing Municipality (grant number 7202229). | The authors declare no competing interests |
| #188-Yao 2020 | This work was supported by a grant from the Shanghai Health Commission (grant no. 201940454). | The authors declare no competing interests |
| #189-Yazdani 2021 | Mashhad University of Medical Sciences, Grant/Award Number: 990637 | The authors declare no competing interests |
| #190-Yeter 2021 | Not stated | The authors declare no competing interests |
| #191-Yildiz 2021 | This research did not receive any specific grant from funding agencies in the public, commercial, or not-for-profit sectors. | The authors declare no competing interests |
| #192-Yilmaz 2021 | Not stated | The authors declare no competing interests |
| #193-Yip 2019 | This study was funded by a Pitch-For-Fund grant administered by Tan Tock Seng Hospital in 2012. | The authors declare no competing interests |

**Supplementary Table 4.** Risk of bias summary figure adapted from Newcastle-Ottawa Scale^[1]^

| **Study ID** | **Representativeness of the cases** | **Selection of Controls** | **Definition of controls** | **Ascertainment of lack of exposure** |
| --- | --- | --- | --- | --- |
| #18-Agarwal 2018 |  |  |  |  |
| #20-Agarwal 2020 |  |  |  |  |
| #21-Agrawal 2016 |  |  |  |  |
| #1-Agrawal 2016 |  |  |  |  |
| #22-Agrawal 2021 |  |  |  |  |
| #28-Aksoy 2022 |  |  |  |  |
| #29-Alis 2022 |  |  |  |  |
| #30-Altinel 2022 |  |  |  |  |
| #32-Asikgarip 2021 |  |  |  |  |
| #31-Asikgarip 2022 |  |  |  |  |
| #34-Azuma 2021 |  |  |  |  |
| #35-Bakthavatsalam 2017 |  |  |  |  |
| #38-Balci 2020 |  |  |  |  |
| #37-Balci 2021 |  |  |  |  |
| #36-Balci 2022 |  |  |  |  |
| #39-Bayram 2022 |  |  |  |  |
| #42-Bousquet 2021 |  |  |  |  |
| #45-Ceri 2022 |  |  |  |  |
| #46-Cevher 2022 |  |  |  |  |
| #47-Ceylanoglu 2022 |  |  |  |  |
| #51-Cheong 2022 |  |  |  |  |
| #52-Chun 2022 |  |  |  |  |
| #53-Cicinelli 2022 |  |  |  |  |
| #54-Damian 2021 |  |  |  |  |
| #55-Dave 2022 |  |  |  |  |
| #56-DeBernardo 2021 |  |  |  |  |
| #62-Durusoy 2021 |  |  |  |  |
| #6-G Giannaccare  2020 |  |  |  |  |
| #13-G Ledesma-gil 2022 |  |  |  |  |
| #71-GulerAlis 2021 |  |  |  |  |
| #75-Hepokur 2021 |  |  |  |  |
| #77-Hwang 2022 |  |  |  |  |
| #78-Inam 2019 |  |  |  |  |
| #80-Iovino 2021 |  |  |  |  |
| #81-Iovino 2022 |  |  |  |  |
| #82-Isik 2022 |  |  |  |  |
| #85-Karasu 2021 |  |  |  |  |
| #91-Kim 2018 |  |  |  |  |
| #90-Kim 2018 |  |  |  |  |
| #98-Kocak 2020 |  |  |  |  |
| #97-Kocak 2021 |  |  |  |  |
| #96-Kocak 2022 |  |  |  |  |
| #101-Krytkowska 2021 |  |  |  |  |
| #109-Liu 2018 |  |  |  |  |
| #110-Loiudice 2021 |  |  |  |  |
| #114-Ma 2022 |  |  |  |  |
| #113-Ma 2022 |  |  |  |  |
| #115-Magesan 2022 |  |  |  |  |
| #9-MG Ersoz  2021 |  |  |  |  |
| #117-Mirshahi 2022 |  |  |  |  |
| #120-Nivison-Smith 2020 |  |  |  |  |
| #121-Obada 2022 |  |  |  |  |
| #124-Ozcaliskan 2020 |  |  |  |  |
| #125-Ozcan 2021 |  |  |  |  |
| #128-Ozcelik-Kose 2021 |  |  |  |  |
| #129-Ozcelik-Kose 2022 |  |  |  |  |
| #131-Ozer 2020 |  |  |  |  |
| #133-Parisi 2020 |  |  |  |  |
| #134-Park 2019 |  |  |  |  |
| #135-Pellegrini 2019 |  |  |  |  |
| #140-Robbins 2021 |  |  |  |  |
| #139-Robbins 2021 |  |  |  |  |
| #141-Sacconi 2022 |  |  |  |  |
| #7-SB Velaga 2020 |  |  |  |  |
| #143-Scarinci 2021 |  |  |  |  |
| #144-SekeryapanGediz 2021 |  |  |  |  |
| #147-Sevik 2022 |  |  |  |  |
| #150-Shrivastav 2022 |  |  |  |  |
| #151-Sidorczuk 2022 |  |  |  |  |
| #153-Simsek 2022 |  |  |  |  |
| #152-Simsek 2022 |  |  |  |  |
| #157-Su 2020 |  |  |  |  |
| #159-Tan 2016 |  |  |  |  |
| #160-Tan 2018 |  |  |  |  |
| #165-Temel 2021 |  |  |  |  |
| #164-Temel 2021 |  |  |  |  |
| #162-Temel 2021 |  |  |  |  |
| #167-Temel 2022 |  |  |  |  |
| #163-Temel 2022 |  |  |  |  |
| #161-Temel 2022 |  |  |  |  |
| #168-Topcuoglu 2021 |  |  |  |  |
| #170-UcgulAtilgan 2023 |  |  |  |  |
| #172-Uslu 2021 |  |  |  |  |
| #173-Viggiano 2022 |  |  |  |  |
| #177-Wang 2021 |  |  |  |  |
| #175-Wang 2021 |  |  |  |  |
| #176-Wang 2022 |  |  |  |  |
| #181-Wei 2018 |  |  |  |  |
| #179-Wei 2019 |  |  |  |  |
| #180-Wei 2022 |  |  |  |  |
| #182-Wu 2021 |  |  |  |  |
| #186-Yang 2022 |  |  |  |  |
| #188-Yao 2020 |  |  |  |  |
| #189-Yazdani 2021 |  |  |  |  |
| #190-Yeter 2021 |  |  |  |  |
| #191-Yildiz 2021 |  |  |  |  |
| #192-Yilmaz 2021 |  |  |  |  |
| #193-Yip 2019 |  |  |  |  |

**Supplementary figure 1. Primary analysis with forest plots of individual studies
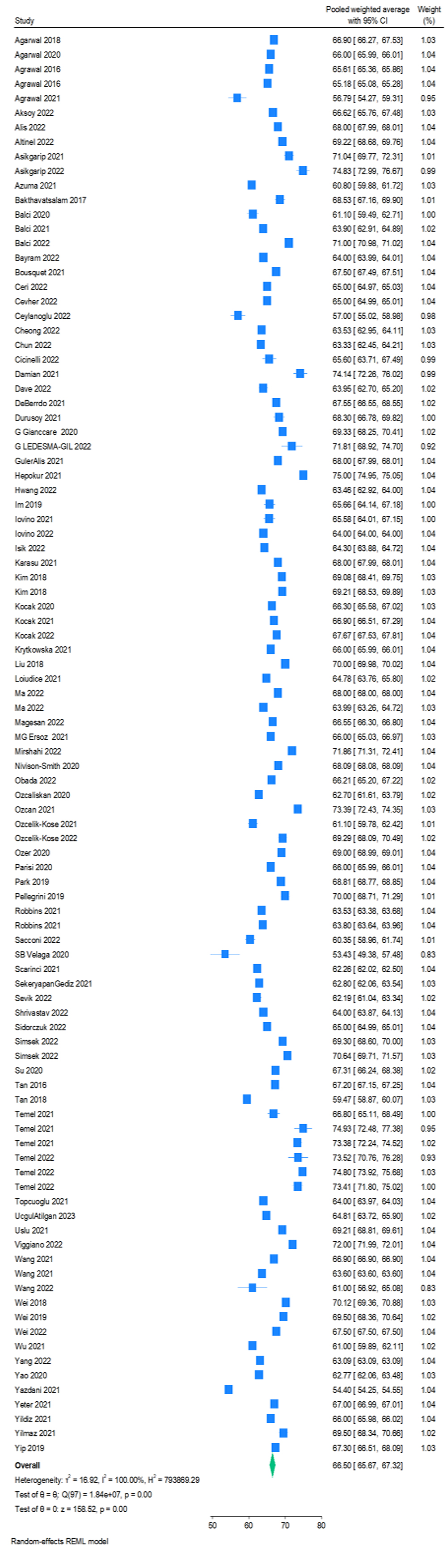
**


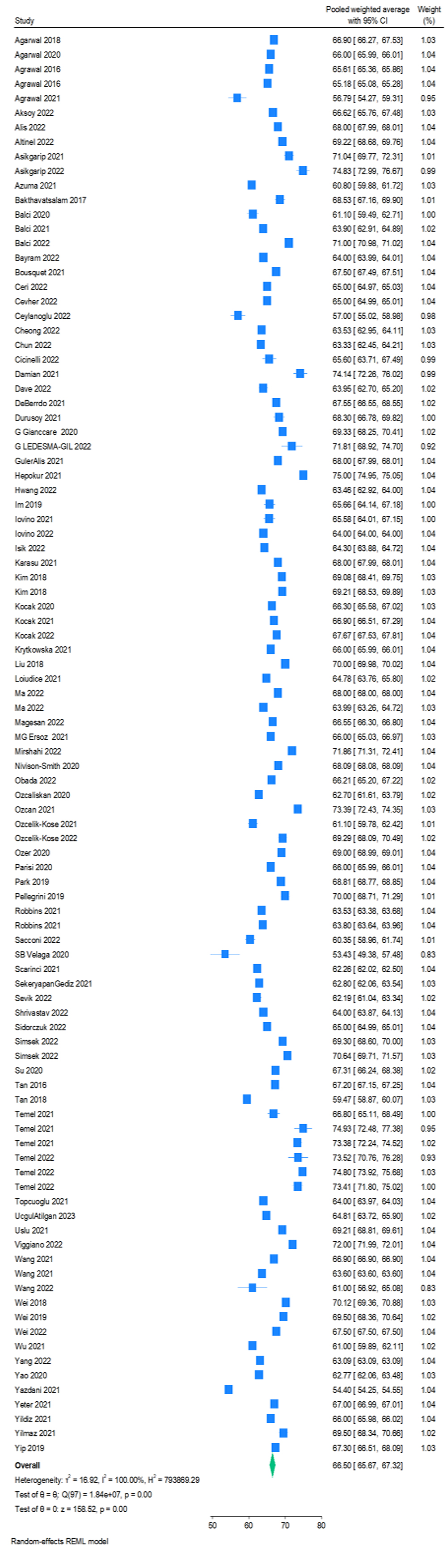


**Supplementary figure 2. CVI’s association with choroidal thickness**

Supplementary Table 5. GRADE assessment of studies in meta-analyses.

GRADE assessment was performed wherein the observational studies start with low quality as baseline^[2,3]^

|  | **Factors that can reduce the quality of the evidence** | | | | | **Factors that can increase the quality of the evidence** | | |  |
| --- | --- | --- | --- | --- | --- | --- | --- | --- | --- |
|  | **Risk of Bias** | **Inconsistency of results** | **Indirectness of evidence** | **Imprecision** | **Publication bias** | **Large magnitude of effect** | **Dose-response effect** | **Plausible confounding reduced effect** | **Overall quality of evidence** |
| **Score** | 0 | 0 | 0 | 0 | 0 | +2 | 0 | +1 | Moderate |
| **Reasoning** | Majority of the studies displayed low risk in ¾ categories | Prediction interval represents narrow margin | Population, and outcome are relevant, Intervention, comparator and comparison are not applicable for control arm | Narrow Confidence Interval for the pooled mean indicated good precision | Publication bias was low as indicated by the Galbraith plot | Pooled weighted mean of the studies with enough studies representing and narrow CI | NA. only control arm included in review | The estimated effect size was not controlled for ethnicity, however the distribution of various ethnic population within studies is likely to have reduced the confounding effects. | ⊕⊕⊕ |

**Supplementary Table 6.** Search strings (using relevant indexed terms where available) for each electronic database, number of results, and eligible articles (including overlaps between databases). Searches were limited to journal articles in English from inception to 1st November 2022

| **Database** | **Search terms** | **Results** | **Eligible studies** |
| --- | --- | --- | --- |
| PubMed | (Choroidal vascularity index[Text]) OR (Choroid vascular index[Text]) AND (Choroid vascularity index [Text] ) OR (choroidal vascular index[Text]) | 359 | 194 |
| Embase (OVID) | (Choroidal vascularity index[Text]) OR (Choroid vascular index[Text]) AND (Choroid vascularity index [Text] ) OR (choroidal vascular index[Text]) | 363 | 3 |
| Total unique studies | | | 197 |

**Supplementary Table 7.** This table lists all the study factors extracted and their role in statistical analysis.

| **Data extracted** | | **Conditions** |
| --- | --- | --- |
| Person and Eye level factors | - Age - Sample size - Axial length - Refractive error - Best corrected visual acuity - Intraocular pressure - Presence of systemic vascular related diseases (whenever available). | These conditions were part of inclusion criteria and were used to analyse regression of the primary outcome variable.  Presence of systemic disease was not considered basis for exclusion or inclusion and was used to aid in subgroup analysis |
| Image level factors | - Optical coherence tomography device and wavelength   - SD OCT (670-870nm)   - Swept Source (1050nm)   - Time of scan ^ß^   - AM-9.00 to 11.59 am   - PM-12.00noon onwards - Imaging mode   - Standard   - EDI - Region of interest (foveally centered, in diameter)   - 1-1.5mm   - 2-3mm   - >3 mm | These conditions were not considered basis for exclusion or inclusion. These were used to aid in subgroup analysis |
| Publication factors | - List of authors - Country of publication - Publication date - Study design - Funding sources - Conflict of interest | These factors were used for the risk of bias analysis |

ß- Data modified to extract relevant details

**Supplementary Table 8.** Questions used for risk of bias assessment of included studies, adapted from the ‘Newcastle-Ottawa Scale for assessing the quality of non-randomized studies in Meta-analyses’. ^[1]^

| **Questions considered** | **Control Selection** | **Categories** |
| --- | --- | --- |
| Representative of the cohort | Was a consecutive or random sample of patients enrolled? | 1. Consecutive recruitment 2. Non-consecutive recruitment |
| Selection of the controls | Could the selection of patients and their site have introduced bias? | 1. Site of recruitment mentioned (Community/ Hospital or University) 2. No description |
| Definition of controls | Did the study adequately describe the healthy status of controls? | 1. No history of disease 2. No description of source |
| Ascertainment of exposure | Did the study describe the method which was utilised to deduce the healthiness of the controls? | 1. Ascertainment method mentioned (Clinical records Clinical assessment / Self-reported) 2. No description |

References:

1. Ga, W. The Newcastle-Ottawa Scale (NOS) for assessing the quality of nonrandomised studies in meta-analyses. in *3rd Symposium on Systematic Reviews: Beyond the Basics, Oxford, UK, 3-5 July 2000* (2000).

2. Guyatt, G. *et al.* GRADE guidelines: 1. Introduction—GRADE evidence profiles and summary of findings tables. *J. Clin. Epidemiol.* **64**, 383–394 (2011).

3. Schünemann, H., Brożek, J., Guyatt, G., Oxman, A., & others. The GRADE handbook. (2013).
